# Advanced Brain-Age in Psychotic Psychopathology: Evidence for Transdiagnostic Neurodevelopmental Origins

**DOI:** 10.3389/fnagi.2022.872867

**Published:** 2022-04-22

**Authors:** Caroline Demro, Chen Shen, Timothy J. Hendrickson, Jessica L. Arend, Seth G. Disner, Scott R. Sponheim

**Affiliations:** ^1^Department of Psychiatry and Behavioral Sciences, University of Minnesota, Minneapolis, MN, United States; ^2^Department of Psychology, University of Minnesota, Minneapolis, MN, United States; ^3^Informatics Institute, University of Minnesota, Minneapolis, MN, United States; ^4^Minneapolis Veterans Affairs Health Care System, Minneapolis, MN, United States

**Keywords:** brain-age, schizophrenia, bipolar, psychosis, advanced aging, neurodevelopment

## Abstract

Schizophrenia is characterized by abnormal brain structure such as global reductions in gray matter volume. Machine learning models trained to estimate the age of brains from structural neuroimaging data consistently show advanced brain-age to be associated with schizophrenia. Yet, it is unclear whether advanced brain-age is specific to schizophrenia compared to other psychotic disorders, and whether evidence that brain structure is “older” than chronological age actually reflects neurodevelopmental rather than atrophic processes. It is also unknown whether advanced brain-age is associated with genetic liability for psychosis carried by biological relatives of people with schizophrenia. We used the Brain-Age Regression Analysis and Computation Utility Software (BARACUS) prediction model and calculated the residualized brain-age gap of 332 adults (163 individuals with psychotic disorders: 105 schizophrenia, 17 schizoaffective disorder, 41 bipolar I disorder with psychotic features; 103 first-degree biological relatives; 66 controls). The model estimated advanced brain-ages for people with psychosis in comparison to controls and relatives, with no differences among psychotic disorders or between relatives and controls. Specifically, the model revealed an enlarged brain-age gap for schizophrenia and bipolar disorder with psychotic features. Advanced brain-age was associated with lower cognitive and general functioning in the full sample. Among relatives, cognitive performance and schizotypal symptoms were related to brain-age gap, suggesting that advanced brain-age is associated with the subtle expressions associated with psychosis. Exploratory longitudinal analyses suggested that brain aging was not accelerated in individuals with a psychotic disorder. In sum, we found that people with psychotic disorders, irrespective of specific diagnosis or illness severity, show indications of non-progressive, advanced brain-age. These findings support a transdiagnostic, neurodevelopmental formulation of structural brain abnormalities in psychotic psychopathology.

## Introduction

Schizophrenia is a debilitating mental disorder, characterized by psychotic symptoms and cognitive impairments, and individuals with the illness often have difficulties functioning in social and community settings. Crucially, it is unclear whether the neuropathology underlying the syndrome is neurodevelopmental or neurodegenerative in nature (Walker et al., 2010; Kochunov and Hong, 2014). Evidence for aberrant neurodevelopment in schizophrenia includes increased rates of adverse events during pregnancy and birth that lead to abnormal prenatal brain development, early signs of cognitive and behavioral abnormalities during childhood, and brain structural abnormalities that are evident during adolescence, either prior to illness onset or early in the course of the illness (Lewis and Levitt, 2002: Rapoport et al., 2005). Nevertheless, schizophrenia, which was originally described as *dementia praecox* [premature dementia; Kraepelin et al. (1971)], has recently been hypothesized to be associated with accelerated cell aging such that normal age-related physiological changes occur at an earlier age. The premature aging hypothesis is supported by evidence of increased rates of cardiovascular and metabolic syndromes (Hennekens et al., 2005; von Hausswolff-Juhlin et al., 2009) and lower cognitive performance in schizophrenia that is similar to older healthy controls (Kirkpatrick et al., 2008).

In the current investigation, we examined whether abnormalities in brain structure associated with schizophrenia are consistent with a pattern of atypical neurodevelopment or premature aging, whether such abnormalities extend beyond schizophrenia to other disorders with psychotic symptoms, and if increased genetic liability for psychotic psychopathology is associated with aberrant brain structure. Because structural brain abnormalities in schizophrenia are widespread and diverse, we used a recently developed brain-age algorithm to quantify and summarize the degree of overall deviation in brain morphology. We further investigated how such deviation is related to symptomatology and cognitive impairments that are central to the disorder.

A neurodevelopmental pattern of abnormalities—where the brain fails to achieve a normative state—is consistent with global structural brain abnormalities that appear early in the course of schizophrenia and are highly heritable (Keshavan et al., 2008). For example, whole brain and total gray matter volumes are reduced in schizophrenia compared to what is seen in typical development (Daniel et al., 1991; Ward et al., 1996; Wright et al., 2000; Shenton et al., 2001; Steen et al., 2006; Gupta et al., 2015; Van Erp et al., 2018). Meta-analyses of first-episode psychosis studies (Steen et al., 2006; Vita et al., 2006) have provided evidence of smaller whole brain and hippocampal volumes as well as larger ventricular spaces in comparison to controls. Also, gray matter perturbations in the absence of medication effects are evident early in the course of the illness (Cahn et al., 2002; Hietala et al., 2003; Szeszko et al., 2003).

To characterize advanced aging of the brain, researchers have developed machine learning tools to quantify the aggregate impact of gray matter loss in a variety of health conditions. One commonly used model estimates a person’s age based on T1-weighted structural imaging data (Franke et al., 2010; Liem et al., 2017). The difference between the model-estimated age and chronological age is the estimated brain-age gap.^1^ The brain-age gap gives an estimate of gray matter abnormalities throughout the brain. Brain-age models generally produce brain-age estimates for people with schizophrenia that are older than true chronological age, by about 2.5–8 years (Koutsouleris et al., 2014; Schnack et al., 2016; Nenadić et al., 2017; Shahab et al., 2019; Truelove-Hill et al., 2020; Constantinides et al., 2022). This pattern extends to early phases of illness, including first-episode psychosis (Kolenic et al., 2018; Hajek et al., 2019), schizophreniform and psychotic disorder not otherwise specified (Shahab et al., 2019), and clinical high risk (Chung et al., 2018) or ultra-high risk (Iftimovici et al., 2020) for psychosis. Despite evidence that abnormalities in brain morphology occur prior to the development of psychosis, the gap between model-estimated and chronological age widens with illness duration such that chronic schizophrenia has the largest gap, followed by recent-onset schizophrenia, and then ultra-high risk for psychosis (Koutsouleris et al., 2014), which could reflect a neurodegenerative process. Importantly, longitudinal data suggest that the brain-age gap may progressively increase over time in schizophrenia (Schnack et al., 2016). Thus, research to date has generated evidence for both neurodevelopmental and neurodegenerative processes in schizophrenia.

Regardless of the origins of aberrant brain morphology in schizophrenia, detection of advanced brain aging may have clinical utility. A large brain-age gap in combination with an early onset of clinical high risk symptoms may predict an insidious onset of psychosis (Chung et al., 2019a,b). Such information could be used in a risk calculator, and possibly be clinically useful in connecting those individuals at highest risk of developing a severe and persistent mental illness with appropriate treatments. However, a critical test for a clinical detection tool is whether it differentiates between individuals who develop psychotic psychopathology from those who do not. Contrasting brain-age of affected and unaffected individuals in a family is one way to appraise the potential for using an index to predict development of the disorder. Before any clinical implementation, however, more research is needed to replicate previous findings, explore diagnostic specificity, and test generalizability to a wider population, as well as understand the influence of confounds (e.g., obesity) to the link between brain-age gap and psychotic illness (Nenadić et al., 2017; Andreou and Borgwardt, 2020).

Whether advanced brain-age is specific to schizophrenia among mental disorders is unclear. There is some evidence for specificity to schizophrenia based on lack of abnormally large age gaps among mixed samples of bipolar disorder with and without psychosis (Nenadić et al., 2017; Shahab et al., 2019), offspring of people with bipolar disorder (Hajek et al., 2019), and non-psychotic major depressive disorder [Besteher et al., 2019; although, see Han et al. (2021)]. However, there is some evidence of larger age gaps among major depressive disorder and borderline personality disorder in comparison to controls, though these gaps are attenuated in comparison to that of the schizophrenia group (Koutsouleris et al., 2014). Structural brain abnormalities such as ventricular enlargement which underlie advanced brain-age estimation do not seem to be specific to schizophrenia, but extend to other psychotic disorders such as psychotic bipolar disorder (Strasser et al., 2005; Keshavan et al., 2008). Further, when comparing bipolar disorder participants with and without a history of psychosis, no difference in brain-age gap was found (Shahab et al., 2019). Based on this previous work, advanced brain-age is most evident in schizophrenia among psychiatric illnesses, but is present to a lesser degree in other mental health conditions; however, few studies to date have specifically compared schizophrenia with other psychotic disorders, including bipolar disorder with psychotic features and schizoaffective disorder. Therefore, it is unknown whether a larger brain-age gap is more generally evident in psychotic psychopathology rather than specific to the diagnostic category of schizophrenia.

In the current study, we examined whether schizophrenia and other psychotic disorders are associated with an advanced brain-age gap. Additionally, we tested the degree to which biological relatives demonstrate brain-age gaps similar to their family members who exhibit psychotic psychopathology. We hypothesized that people with schizophrenia would demonstrate the most advanced brain-age followed by other psychotic disorders and then relatives, who were expected to show attenuated abnormality to people with psychotic disorders (PwP). We also investigated the relationship of brain-age gap with cognitive impairment, functioning, and symptom severity. In order to examine whether brain-age gap suggests neurodegeneration, we conducted exploratory analyses in a subgroup of participants with longitudinal data. Finally, we tested for group differences in gray matter volume, cortical thickness, and surface area in order to approximate specific brain regions with structural deficits that lead to an advanced brain-age gap estimated by the model.

## Materials and Methods

### Participants

Data were collected as part of two family studies focusing on psychosis. Study 1 recruited adults aged 18–65, including people with major mental illness (schizophrenia, schizoaffective disorder, bipolar I disorder without psychotic features, and bipolar I disorder with psychotic features), their first-degree biological relatives, and unrelated healthy controls. Only patients with a history of psychotic symptoms were included in primary analyses, though a separate analysis was completed comparing participants with bipolar I disorder with and without psychotic features (see below). Study 2, the Psychosis Human Connectome Project, recruited people aged 18–65 with a psychotic disorder (schizophrenia, schizoaffective disorder, or bipolar I disorder with psychotic features), their first-degree biological relatives (aged 18–69), and unrelated healthy controls. Exclusion criteria for study 2 have been described previously (Demro et al., 2021) and match those of study 1. In total, 332 participants (48 from study 1; 284 from study 2) were included in primary analyses: 163 participants with psychosis (105 schizophrenia, 17 schizoaffective, 41 bipolar I disorder with psychotic features), 103 first-degree biological relatives (57 siblings, 35 parents, 11 offspring), and 66 controls. Of the 103 relatives, 63 were related to a person with schizophrenia, seven to a person with schizoaffective disorder, and 33 to a person with bipolar I disorder with psychotic features. Forty-two participants (nine controls, 13 relatives, 20 PwP) completed both studies (within 2 weeks–4 years; mean time between scans = 633.55 days, *SD* = 415.10 days), allowing for exploratory analyses of longitudinal data. For the primary analyses, study 2 data were used for participants who completed both studies. An additional 15 individuals with bipolar I disorder without psychosis and seven relatives of people with such a diagnosis were included in exploratory analyses. Groups were matched on basic demographic characteristics as much as possible during study enrollment. For descriptive statistics of the full sample, see Table 1.

**TABLE 1:** Demographic and clinical characteristics.

	Controls *n* = 66	Relatives *n* = 103	PwP *n* = 163	Statistic
Mean age in years (*SD*)	41.16 (12.69)	45.93^c^ (14.24)	40.18^c^ (12.65)	*F*_(2,329)_ = 6.26, *p* = 0.002
Female sex	35 (53.0%)	65^c^ (63.1%)	68^c^ (41.7%)	χ^2^_(2,332)_ = 11.75, *p* = 0.003
Racial/ethnic minority identity	6^a^ (9.1%)	13^c^ (12.6%)	52^ac^ (31.9%)	χ^2^_(2,332)_ = 21.36, *p* < 0.001
Parent education	5.77 (1.18)	5.56 (1.22)	5.39 (1.28)	*H*_(2)_ = 4.44, *p* = 0.108
Participant education (years)	16.08^ab^ (2.27)	15.10^bc^ (2.30)	13.96^ac^ (2.02)	*F*_(2,329)_ = 24.62, *p* < 0.001
BMI (kg/m^2^)	26.15^a^ (5.18)	28.39^c^ (5.93)	31.25^ac^ (7.40)	*F*_(2,328)_ = 15.60, *p* < 0.001
BPRS total	27.03^ab^ (3.51)	32.41^bc^ (6.84)	44.87^ac^ (12.45)	*F*_(2,329)_ = 100.44, *p* < 0.001
SPQ total	7.85^ab^ (7.51)	14.96^bc^ (13.09)	31.14^ac^ (16.33)	*F*_(2,327)_ = 81.12, *p* < 0.001
PID-5 negative affect	0.89^a^ (0.32)	0.98^c^ (0.37)	1.31^ac^ (0.42)	*F*_(2,320)_ = 35.82, *p* < 0.001
WAIS IQ	109.09^ab^ (12.28)	101.88^bc^ (11.35)	97.40^ac^ (12.15)	*F*_(2,328)_ = 22.81, *p* < 0.001

Groups that share a superscript reflect a significant (p < 0.05) pairwise comparison; Participant racial/ethnic identities were as follows, in order of group (controls/relatives/PwP): 90.9/87.4/68.1% White, 4.5/6.8/20.9% Black, 1.5/2.9/3.7% Latino/a, 1.5/1.0/3.1% Asian/Asian American, 0/0/0.6% Native American, 1.5/1.9/3.7% Other; Number of participants who were missing data: nine on parent education, two on SPQ and one on WAIS IQ; Parent education = highest of either parent’s level of education achieved, coded on an ordinal scale: 1 = 7th grade or less, 2 = 7th–9th grade, 3 = 10th–12th grade, 4 = high school graduate/GED, 5 = partial college/vocational/technical/RN, 6 = 4 year college/university graduate, 7 = graduate degree; BMI = body mass index calculated as [weight/(height * height)] and then multiplied by 703 to convert to metric units; BPRS = Brief Psychiatric Rating Scale (minimum score = 24); SPQ = Schizotypal Personality Questionnaire; PID-5 = Personality Inventory for DSM-5; WAIS IQ = estimated from Wechsler Adult Intelligence Scale.

### Clinical Assessment

The Structured Clinical Interview for DSM-IV-TR disorders (First et al., 2002) was used to assess clinical diagnosis and estimate Global Assessment of Functioning (GAF) for participants in both studies. The Brief Psychiatric Rating Scale [BPRS; Ventura et al. (2000)] was administered to all participants as a broad measure of psychiatric symptom severity. Participants with psychosis were additionally assessed with the Scale for the Assessment of Negative/Positive Symptoms [SANS/SAPS; Andreasen (1984, 1989)]. This measure provides an estimate of current psychotic symptom severity. The Schizotypal Personality Questionnaire [SPQ; Raine (1991)] was used to capture subtle psychosis-like traits. Similarly, to capture variation in propensity for dysregulated negative emotional states the trait domain “negative affect” of the Personality Inventory for DSM-5 (PID-5) was used in analyses (Longenecker et al., 2020). Cognitive functioning was assessed using subscales of the Wechsler Adult Intelligence Scale (WAIS; Wechsler, 2008) to yield an estimate of IQ.

### Neuroimaging Data Acquisition

Study 1 collected structural Magnetic Resonance Imaging (MRI) data using a 10-min T1-weighted MPRAGE sequence (TE = 2.12 ms, TR = 2,400 ms, flip angle = 8, resolution = 256) on a 3 Tesla Siemens Prisma scanner using a 32 channel head coil. Imaging data for study 2 were collected on a separate Siemens 3 Tesla Prisma scanner with a Siemens 32 channel head coil. An 8-min HCP T1w MPRAGE (TE = 1.81/3.6/5.39/7.18 ms, TR = 2,500 ms, flip angle = 8, resolution = 256) with volumetric navigators for real-time motion correction was performed. Both studies completed MRI scanning at the Center for Magnetic Resonance Research of the University of Minnesota.

### Neuroimaging Data Processing

The T1-weighted DICOM data was converted to NIfTI by following the standardized brain imaging data structure (BIDS) format (Gorgolewski et al., 2016). The BIDS-organized NifTI data was then processed through the Brain-Age Regression Analysis and Computation Utility Software [BARACUS; Liem et al. (2017)] using a containerized version of the software (Liem and Gorgolewski, 2017). The BARACUS container was downloaded locally and used to produce the BARACUS model estimate of brain-age. The BARACUS container includes all functionality and software dependencies to take raw NIFTI data, run the FreeSurfer processing, and calculate the brain-age estimate based on the BARACUS model. We used BARACUS version 1.1.2. The BARACUS framework uses previously trained machine learning based prediction models to estimate the age of a participant based on their anatomical data. To perform the prediction, BARACUS utilizes metrics from the cortical reconstruction and volumetric segmentation tool FreeSurfer v5.3.0 (Dale and Sereno, 1993; Sled et al., 1998; Dale et al., 1999; Fischl et al., 1999a,b, 2001, 2002, 2004a,b; Fischl and Dale, 2000; Rosas et al., 2002; Kuperberg et al., 2003; Salat et al., 2004; Segonne et al., 2004; Desikan et al., 2006; Han et al., 2006; Jovicich et al., 2006; Segonne et al., 2007; Reuter et al., 2010, 2012; Reuter and Fischl, 2011) such as cortical thickness, cortical surface area, and subcortical volumes. The BARACUS model was trained and implemented in a two-level approach. The first level predicted ages with a support vector regression model (SVR) (Drucker et al., 1996) from each FreeSurfer metric separately. The second level stacked all of the SVR models from the first level with a random forest (RF) model (Breiman, 2001). Previous research suggests that generating a predictive model with this two-level strategy with neuroimaging data produces prediction with smaller variability (Rahim et al., 2016). The present analysis used the previously trained BARACUS model “Liem (2016)__OCI_norm,” which was trained on 1,166 participants (566F/600M, age: μ = 59.1, σ = 15.2, range = 20–80) with no objective cognitive impairment (OCI), because it most closely matched the demographics of our sample.

### Brain-Age Gap Estimate

The brain-age gap estimate is the difference between a person’s chronological age and their model-estimated age. The model-estimated age was calculated using the publicly available BARACUS model. We used a corrected version of brain-age gap [see Smith et al. (2019)] because chronological age correlates with brain-age gap, leading to overestimation of age in younger people and underestimation in older people due to regression to the mean (Le et al., 2018). In order to correct for this, we computed the age gap by subtracting chronological age from the model-estimated age. We then fit a regression line to our data, predicting the age gap from chronological age and saving the unstandardized residuals. The residual values were then used for subsequent analyses and are hereafter referred to as brain-age gap estimates.^2^

### Statistical Analysis

Data analyses were completed using SPSS version 25 and R version 4.1.1. We conducted multilevel modeling to examine whether participants were nested due to family relatedness. We then used multilevel modeling to assess group differences in brain-age gap, accounting for family relatedness. Scanner type was used as a covariate in analyses to control for possible effects related to each study using a different MRI scanner. Additional covariates included race, sex, and BMI, as groups differed on these variables and previous studies, as well as our own analyses, suggest that these variables are associated with brain-age gap (Nenadić et al., 2017; Kolenic et al., 2018). We first tested for a main effect of group on brain-age gap by comparing healthy controls, relatives, and PwP. We then split the group of PwP into subgroups reflecting clinical diagnosis (schizophrenia, schizoaffective disorder, bipolar disorder with psychotic features). We conducted multilevel modeling (controlling for scanner, race, sex, and BMI) to assess the relation between brain-age gap and various clinical and cognitive measures, using False Discovery Rate (FDR) to correct for multiple comparisons.

In terms of exploratory analyses, we used linear regression to examine relations between brain-age gap and clinical and cognitive measures among subgroups of participants, controlling for scanner, race, sex, and BMI. We used mixed analysis of variance (ANOVA) to examine group differences in brain-age gap over time for a subset of participants who completed both study 1 and study 2. Using a subset of participants from study 1 that are not included in primary analyses, we conducted an analysis of covariance (ANCOVA; controlling for sex, race, and BMI) to test for group differences between 15 individuals with bipolar I disorder without psychosis, seven relatives of people with such a diagnosis, and the other groups previously described. Finally, we used ANCOVA to test for group differences in cortical thickness, cortical surface area, and subcortical volume. We entered cortical thickness, cortical surface area, and subcortical volume measures for all regions as dependent variables, group status (control, relative, PwP) as the independent variable, and sex, age, scanner, and estimated total intra-cranial volume as covariates. We corrected for multiple comparisons using FDR.

## Results

Demographic and clinical characteristics of the sample are provided in Table 1. Groups differed in terms of age, sex, and race (see Table 1) largely due to relatives who were older, more female, and more White than PwP. Controls and PwP had similar demographic characteristics (i.e., no differences with the exception of race) and there were no group-by-age [*F*_(70,210)_ = 1.26, *p* = 0.109], group-by-sex [*F*_(2,326)_ = 0.43, *p* = 0.653] or group-by-race [*F*_(8,316)_ = 0.62, *p* = 0.760] interactions in predicting brain-age gap. As in previous studies, obesity [defined as BMI > 30 kg/m^2^; Weir and Jan (2021)] was associated with more advanced brain-age [*F*_(1,330)_ = 7.23, *p* = 0.008] but there was no group-by-obesity status interaction in predicting brain-age gap [*F*_(2,326)_ = 1.96, *p* = 0.143]. Even though only 50 of the 163 PwP were related to one or more relatives in the study, and families in our sample were small (the modal PwP-relative family contained one relative), results of multilevel modeling suggest that brain-age gap data were nested within families. Primary analyses were conducted with the following covariates: sex, race, BMI, family relatedness, and scanner.

**FIGURE 1:**
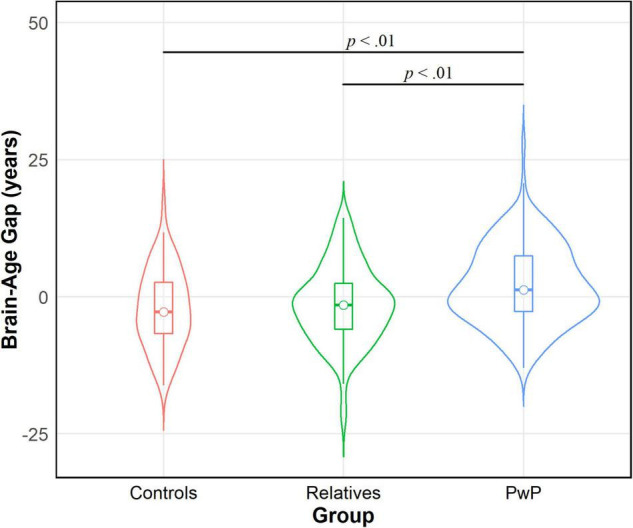
Violin density plot of group comparison on brain-age gap. People with psychotic disorders (PwP) demonstrated a greater estimated brain-age than chronological age (i.e., brain-age gap) in contrast to biological relatives of people with psychotic psychopathology and healthy controls.

**FIGURE 2:**
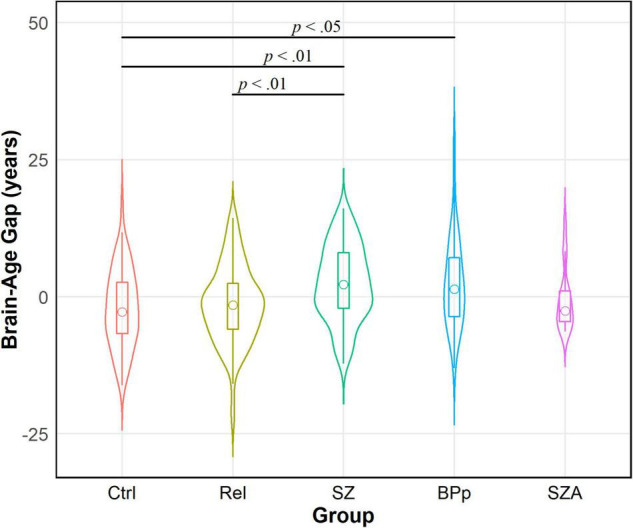
Violin density plot of brain-age gap across diagnostic groups within people with psychotic disorders (PwP). Schizophrenia (SZ) and bipolar I disorder with psychotic features (BPp) groups demonstrated larger brain-age gaps than healthy controls (Ctrl). There were no differences in brain-age gap between the forms of psychotic disorders. Relatives (Rel) had smaller brain-age gaps than SZ; relatives did not differ from BPp, schizoaffective (SZA), or controls. Sample size for SZA is small and interpretation requires caution.

**FIGURE 3:**
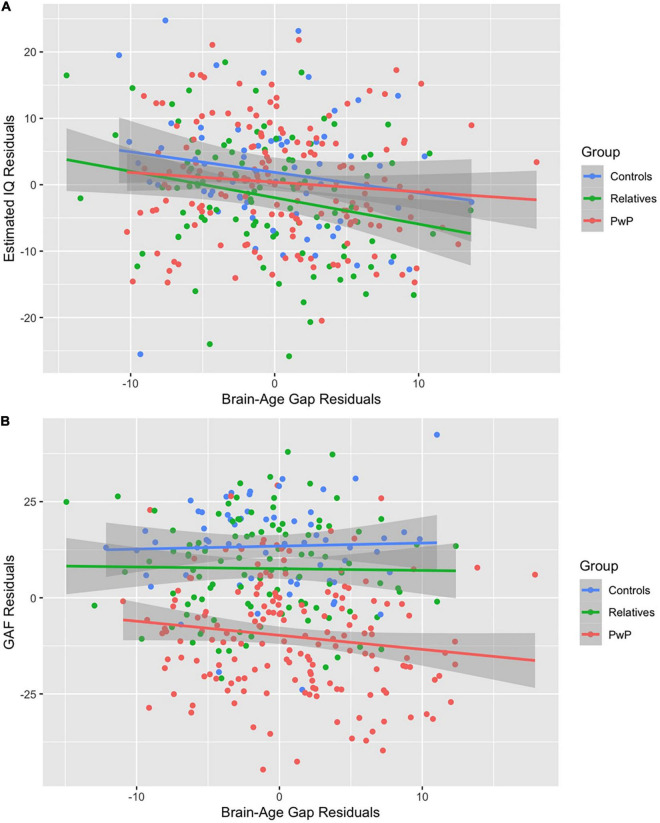
**(A,B)** Correlations among the full sample. Lower IQ **(A)** and lower Global Assessment of Functioning **(B)** predicted larger brain-age gap after adjusting for covariates.

We tested our hypothesis that people with psychotic disorders (PwP), and to a lesser degree their first-degree biological relatives, would demonstrate advanced brain-age compared to healthy controls. The results of multilevel modeling indicated that participant group predicted brain-age gap after accounting for sex, race, BMI, family relatedness, and scanner [χ^2^_(2,332)_ = 17.95, *p* < 0.001]. Specifically, PwP demonstrated a larger brain-age gap compared to their biological relatives [*p* = 0.001] and healthy controls [*p* = 0.002], indicating that the estimated brain-age of individuals with a history of psychosis was further beyond their chronological age than the other groups (see Figure 1). In order to examine whether the particular form of psychotic psychopathology was relevant, we compared subgroups of PwP along clinical diagnostic boundaries. A multilevel model showed that a five-category diagnostic group variable (see Figure 2) predicted brain-age gap when controlling for sex, race, BMI, family relatedness, and scanner [χ^2^_(4,332)_ = 20.26, *p* < 0.001]. Pairwise comparisons indicated that people with schizophrenia and bipolar I disorder with psychotic features demonstrated larger brain-age gaps compared to controls (*p* = 0.008 and *p* = 0.049, respectively; Tukey-corrected), with no group differences between any of the psychotic disorders (*p* > 0.584; see Figure 2). Contrary to hypotheses, biological relatives of PwP failed to have greater brain-age gaps than healthy controls (*p* = 0.990; see Supplementary Material for more details). A multilevel model was run to determine whether IQ and community functioning predicted brain-age gap in the full sample (with scanner, race, sex, BMI, and family relatedness as variables of non-interest in the model). The results of this model indicated that lower IQ (*p* = 0.007), lower functioning (*p* = 0.003), and female sex (*p* < 0.001; see Figure 3) predicted greater brain-age gap.

In exploratory analyses, we examined these relations among subgroups of participants. Among relatives of PwP, multiple regression was run to predict brain-age gap from IQ, community functioning (GAF score), schizotypal symptoms (SPQ total), and emotion dysregulation (PID-5 negative affect), maintaining scanner, race, sex, and BMI as variables of non-interest in the model. Among relatives, lower IQ (*p* = 0.028), lower SPQ (*p* = 0.032), and study 1 scanner (*p* = 0.040) predicted larger brain-age gap [*F*_(8,89)_ = 3.39, *p* = 0.002, *R*^2^ = 0.233]. Since scanner was entered as a variable of non-interest, and there were no group differences to suggest disproportionate assessment by scanner type [χ^2^_(2,332)_ = 4.56, *p* = 0.102], we did not further interpret the contribution of scanner in this analysis. Among PwP, multiple regression with IQ, community functioning, current symptom severity (BPRS total), duration of psychotic illness, and current antipsychotic medication dosage [quantified using chlorpromazine equivalents, see Leucht et al. (2016)] failed to predict brain-age gap, with scanner, race, sex, and BMI entered as covariates [*F*_(9,122)_ = 1.74, *p* = 0.086, *R*^2^ = 0.114]. These results suggest that larger brain-age gap in biological relatives is associated with lower cognitive functioning, while in PwP, brain-age gap is not a marker of current cognitive functioning, symptoms, medication load, or time since disorder onset. This is consistent with brain-age gaps reflecting developmental abnormalities, rather than neurodegeneration, of brain structure in individuals with psychotic disorders.

**FIGURE 4:**
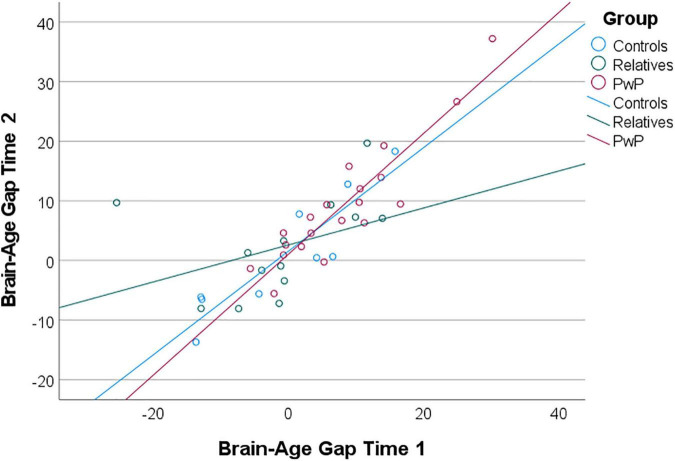
Scatterplot of uncorrected brain-age gap scores from exploratory longitudinal analysis. Brain-age gap scores increased over time for all groups; groups did not differ in change in brain-age gap over time (i.e., brain-age acceleration).

Additional exploratory analyses included a longitudinal examination of brain-age gap among the subgroup of participants who completed both study 1 and study 2. The intraclass correlation coefficient between predicted brain-age scores from the study 1 and study 2 scans was 0.90 (95% CI: 0.81–0.95), suggesting good reliability and stability. A mixed ANOVA controlling for amount of time between scans 1 and 2 revealed that brain-age gap scores increased over time [*F*_(1,38)_ = 4.15, *p* = 0.049], but there was no interaction to suggest a group difference in changes in brain-age gap over time [*F*_(2,39)_ = 0.49, *p* = 0.615; see Figure 4]. In terms of years of brain-age gap reflected in the unadjusted values, PwP increased from an average brain-age gap of 7.94 years (*SD* = 9.03) at the first scan to 9.08 years (*SD* = 10.01) at the follow-up scan; controls increased from -0.75 (*SD* = 10.73) to 0.89 (*SD* = 10.33) years; and relatives increased from -1.35 (*SD* = 10.66) to 2.18 (*SD* = 8.27) years. The brain-age acceleration rate was calculated as the change in uncorrected brain-age gap over the change in chronological age. Aging accelerated at a rate of 2.56 years per chronological year (*SD* = 10.76) for PwP, 2.21 years per chronological year (*SD* = 4.62) for controls, and 1.54 years per chronological year (*SD* = 11.61) for relatives.

In an exploratory analysis to specifically examine the dependency of brain-age gap on history of psychosis, we compared participants with bipolar I disorder without a history of psychosis to the other groups. We found an effect of group on brain-age gap [*F*_(4,163)_ = 4.16, *p* = 0.003] such that participants with bipolar I disorder without psychotic features, surprisingly, had a greater brain-age gap in comparison to bipolar I disorder participants with psychotic features (*p* = 0.029), biological relatives of people with bipolar I disorder with (*p* = 0.003) and without psychotic features (*p* = 0.003), and healthy controls (*p* < 0.001). Although based on a small sample, this effect suggests that other factors beyond psychosis are related to altered brain morphology in bipolar disorder.

In order to investigate which brain regions may be driving brain-age gap findings, we compared groups on cortical thickness and surface area as well as subcortical volume. We found an effect of group in a range of regions when controlling for sex, scanner, age, and intra-cranial volume (see Supplementary Material). Briefly, groups differed in frontal regions (bilateral: caudal middle frontal, lateral orbitofrontal, paracentral, pars opercularis, pars orbitalis, pars triangularis, precentral, superior frontal; left medial orbitofrontal; and right rostral middle frontal), parietal regions (bilateral: inferior parietal, isthmus cingulate, precuneus, postcentral, superior parietal, supramarginal; and right posterior cingulate), temporal regions (bilateral: inferior temporal; fusiform; middle temporal, superior temporal, temporal pole, transverse temporal; and right parahippocampal), and occipital regions (bilateral: lateral occipital, lingual, pericalcarine; and left cuneus) as well as the right banks of superior temporal sulcus (see Supplementary Table 1). People with psychotic disorders had lower cortical thickness, surface area, and/or subcortical volume values than controls and relatives in all these regions; relatives had lower values than controls in only a few regions (surface area in left: lateral orbitofrontal, medial orbitofrontal, middle temporal, and pars opercularis regions; left transverse temporal cortical thickness; and right pericalcarine surface area; see Supplementary Table 1).

## Discussion

Our analysis revealed evidence of advanced brain-age (quantified by a larger gap between chronological and model-estimated age) for individuals with a primary psychotic disorder as well as people with bipolar I disorder with a history of psychotic symptoms. There were no differences in brain-age gap across the types of psychotic disorders, indicating that advanced brain-age is not diagnostically specific in our sample. Biological first-degree relatives, unaffected and affected, demonstrated model-estimated ages that did not differ from their chronological age and did not have an intermediate brain-age gap compared to people with psychosis and healthy controls. Thus, advanced brain-age may possess clinical utility for identifying individuals within a family who are likely to develop psychosis. Exploratory analyses of a subset of individuals with longitudinal data revealed that over an average period of 21 months, psychotic psychopathology failed to be associated with acceleration in brain aging. This supports the assertion that excessive advance in brain-age occurs earlier in the disorder and could reflect neurodevelopmental abnormalities.

Our findings support the presence of an abnormal neurodevelopmental process in psychotic disorders. Brain-age gap was unrelated to duration of psychotic illness, antipsychotic load, or current symptom severity, suggesting that early disruptions to brain development—rather than current clinical severity—are indicative of advanced brain-age. Based on our longitudinal analyses, the large brain-age gap that we observed in schizophrenia and bipolar disorder does not seem to worsen over time. Morphological abnormalities underlying advanced brain aging likely occur early in the course of illness without progressing throughout the lifespan, thus reflecting neurodevelopmental processes [see Shahab et al. (2019)]. However, it is possible that our follow-up period of 21 months on average was too short to observe accelerated atrophy and that there may exist subgroups of persons with psychosis for whom neurodegenerative processes are implicated (Murray et al., 1992).

The current study involved a transdiagnostic sample of individuals with a history of psychotic symptomatology that allowed us to directly compare across diagnostic boundaries. Our primary findings suggest that the presence of psychotic psychopathology, regardless of specific diagnostic category, is implicated in advanced brain-age. Exploratory analysis revealed that advanced brain-age is evident in bipolar disorder regardless of a history of psychosis, which suggests that there are additional factors that affect brain structure in bipolar disorder. However, given the small sample, future studies would be more informative if they included more individuals with bipolar disorder who are classified based on their history of psychosis. The utility of examining brain-age gaps across different psychotic disorders lies in informing transdiagnostic, biologically-based classification. Classifying patients according to biological rather than clinical parameters can identify specific pathophysiological processes that contribute to the manifestation of psychotic symptoms and perhaps explain the heterogeneity within diagnoses (Clementz et al., 2016). Brain-age gap could characterize individual differences in overall brain morphology within psychotic disorders, which may improve the ability to predict the development and course of psychotic psychopathology (Cole et al., 2019). Our findings add to the growing literature by demonstrating cross-disorder associations between brain-age gap and IQ and community functioning. Specifically, we found among biological relatives that lower general cognitive performance predicted greater brain-age gap, suggesting that advanced brain-age could be a measure of subtle effects of genetic liability for psychosis that is more sensitive than the clinical presentation of psychotic psychopathology.

Our study provides further evidence of an association between lower cognitive performance and advanced brain-age. However, the association between advanced brain-age and lower cognitive functioning in our sample appears not to depend on the presence of clinically significant psychopathology. Similarly, lower cognitive performance has been associated with more advanced brain-age gap in healthy controls (Liem et al., 2017; Richard et al., 2018, 2020; Elliott et al., 2021) and in adults with various medical conditions, such as HIV (Kuhn et al., 2018), Alzheimer’s disease (Franke and Gaser, 2012), and TBI (Cole et al., 2015). Within psychotic psychopathology, cognitive performance has been associated with cortical thickness throughout the brain in both schizophrenia and bipolar disorder (Shahab et al., 2019) and structural connectivity in schizophrenia (Yeo et al., 2016), and evidence suggests that different brain regions are related to cognitive function for people with schizophrenia versus controls (Hanford et al., 2019). Further, people with schizophrenia can be classified according to cognitive deficits in specific domains, which correspond to unique structural brain alterations (Geisler et al., 2015).

In terms of brain morphology, our analyses revealed widespread gray matter perturbations among people with psychotic disorders. Brain-age provides an estimate of the aggregate impact of such gray matter deficits. Previous brain-age research has also linked larger brain-age gap with lower gray matter volume: in the prefrontal cortex in typical development (Truelove-Hill et al., 2020), throughout the brain among first-episode psychosis patients (Hajek et al., 2019), and in the left temporal and insular cortices as well as the left frontal and parietal lobes in schizophrenia spectrum disorders (Shahab et al., 2019). These regions are involved in higher-order processing and highly implicated in psychotic disorders. We found evidence of gray matter abnormalities throughout the brain for people with psychosis, overlapping with previous research. Finally, schizophrenia is associated with physical health issues, including cardiovascular disease, obesity, and metabolic syndrome (von Hausswolff-Juhlin et al., 2009). Such comorbid physical conditions require more hospitalizations (Šprah et al., 2017), further increasing the emotional and financial burden of the disorder and contributing to a shorter life span for people with schizophrenia. This increased mortality, in combination with evidence of impaired cognitive performance and abnormal brain development, may well be manifestations of compromised cellular health. The brain-age gap offers a summary value that provides an estimate of the degree to which abnormalities in brain morphology could be characterized using a cell aging algorithm.

There are several strengths of our study. First, the clinical sample included people with a range of psychotic disorders and therefore allowed for transdiagnostic examination of the brain-age gap model. Second, the study included biological first-degree relatives, which allowed for the testing of hypotheses related to genetic loading. Third, the sample size was relatively large for a single site study. Fourth, we used a corrected version of the brain-age gap to address the correlation between chronological age and brain-age gap, which leads to an overestimation of age in younger people and underestimation in older people due to regression to the mean (Le et al., 2018; Smith et al., 2019). The correlation suggests that chronological age can confound the relationship between brain-age gap and variables of interest, yielding spurious correlations. Thus, previous studies that did not correct for this statistical dependency should be interpreted with caution. Much of the data from the current study will be publicly available to allow other investigators to examine brain morphology in psychosis.

There are also some limitations of our study, including using a brain-age gap model that is based on only structural T1-weighted neuroimaging data, rather than incorporating functional data. Previous findings suggest that multimodal data, combining both anatomical and whole-brain functional connectivity information, improves brain-based age prediction in healthy controls (Liem et al., 2017). Another limitation is that our current findings are based on two studies that used different MRI scanners, which limited statistical power in our analyses as we had to covary for scanner type in our models. Further, because the two time points were collected on different scanners, results of longitudinal analyses are considered exploratory and require replication. Our sample was more diverse than the training sample in terms of racial identity and our groups differed on this variable, which is a potential limitation for the effectiveness of the brain-age model as acknowledged by the BARACUS authors (Liem and Gorgolewski, 2017). Body mass index was calculated from body measurements for a subset of participants, whereas it was based on participants’ self-reported estimates of height and weight for all study 1 participants (body measurements were not part of the study 1 protocol) and for 25 study 2 participants (body measurements were not completed during the COVID-19 pandemic). Thus, our BMI variable may contain additional measurement error. However, the results of our group comparisons did not change when we controlled for body mass index. Finally, psychotic disorders have high rates of comorbidity with, for example, mood episodes, trauma, and metabolic health issues. There is a body of research documenting larger brain-age gap among such conditions, including major depressive disorder (Han et al., 2021), post-traumatic stress disorder (Liang et al., 2019; Clausen et al., 2022), and obesity, which co-occurs with both psychosis and cognitive impairment (Bora et al., 2017; Kolenic et al., 2018). Importantly, certain medications, such as those to treat obesity and bipolar disorder, may have neuroprotective effects [see Kolenic et al. (2018)]. These findings highlight the need to understand more about potential confounding factors to advanced brain-age in psychotic psychopathology, not all of which we could address in the current study.

Overall, our study revealed evidence of advanced brain-age in schizophrenia and bipolar disorder. Interestingly, analysis of a subset of individuals with longitudinal data failed to provide evidence of accelerated brain aging in psychotic psychopathology. This is consistent with early, and perhaps neurodevelopmental, neural abnormalities. Relatives of people with psychosis demonstrated an association between brain-age and cognitive performance, suggesting that lower cognitive function in individuals with genetic liability for psychosis may be tied to cellular abnormalities that result in aberrant brain morphology.

## Data Availability Statement

The datasets presented in this article are not readily available because the data from study 2 will be made publicly available through the NIMH Data Archive. Requests to access the datasets should be directed to hodgem@wustl.edu.

## Ethics Statement

The studies involving human participants were reviewed and approved by the Institutional Review Board at the University of Minnesota. The patients/participants provided their written informed consent to participate in this study.

## Author Contributions

SS and SD contributed to the conception and design of the study. CD, CS, TH, and JA contributed to the acquisition, analysis, and interpretation of the data. TH processed the study neuroimaging data with machine-learning based software. CD and CS organized the database and performed the statistical analysis. CD wrote the first draft of the manuscript. CD, TH, and SS wrote sections of the manuscript. SS provided supervision throughout the study and contributed to all stages of the manuscript. All authors contributed to manuscript revision and approved the submitted version.

## Author Disclaimer

The contents do not represent the views of the U.S. Department of Veterans Affairs or the United States Government.

## Conflict of Interest

The authors declare that the research was conducted in the absence of any commercial or financial relationships that could be construed as a potential conflict of interest.

## Publisher’s Note

All claims expressed in this article are solely those of the authors and do not necessarily represent those of their affiliated organizations, or those of the publisher, the editors and the reviewers. Any product that may be evaluated in this article, or claim that may be made by its manufacturer, is not guaranteed or endorsed by the publisher.

## Footnotes

1 Brain-age gap here is the same construct as Brain Age Gap Estimate (BrainAGE), as it is sometimes referred to in this literature.

2 This type of corrected brain-age gap estimate is sometimes referred to as “BrainAGER” (Brain Age Gap Estimate Residualized) scores in the literature.
