# Supplementary material for: Advanced Brain-Age in Psychotic Psychopathology: Evidence for Transdiagnostic Neurodevelopmental Origins
Source: Front Aging Neurosci. 2022 Apr 22;14:872867. doi: 10.3389/fnagi.2022.872867 (PMC9074783; doi:10.3389/fnagi.2022.872867)
Supplement: Supplementary file 1 [file Data_Sheet_1.PDF]

**Supplementary Material**

## Supplementary Results

Biological relatives of PwP did not have significantly larger brain age gap than healthy controls (*p*=.990), regardless of type of psychotic disorder for which they carry genetic liability (whether they are a relative of someone with schizophrenia vs. schizoaffective disorder vs. BPp; all *p*>.084), type of relation (siblings vs. parents vs. offspring; all *p*>.579), and presence of own mental health diagnosis (*n*=57, all *p*>.425).

**Supplementary Table S1.** Group differences on cortical thickness, cortical surface area, and subcortical volume by region.

| Brain region | Controls | Relatives | PwP | *F* | *p* | FDR *p* |
|---|---|---|---|---|---|---|
| ***Cortical Thickness*** | | | | | | |
| L. Bankssts<br>R. | 2.44<br>2.60[a] | 2.41[c]<br>2.51[c] | 2.46[c]<br>2.59[ac] | 1.56<br>8.46 | .211<br><.001 | .278<br>.003 |
| L. Caudal anterior cingulate<br>R. | 2.63<br>2.45 | 2.58<br>2.44 | 2.61<br>2.49 | 1.53<br>1.12 | .218<br>.327 | .282<br>.381 |
| L. Caudal middle frontal<br>R. | 2.56<br>2.55[a] | 2.53<br>2.48 | 2.60<br>2.55[a] | 3.97<br>10.25 | .020<br><.001 | .046<br>.001 |
| L. Cuneus<br>R. | 1.89<br>1.90 | 1.85[c]<br>1.87[c] | 1.91[c]<br>1.92[c] | 4.11<br>2.39 | .017<br>.093 | .042<br>.142 |
| L. Entorhinal<br>R. | 3.35<br>3.59 | 3.28[c]<br>3.56 | 3.41[c]<br>3.65 | 3.44<br>1.24 | .033<br>.290 | .065<br>.357 |
| L. Frontal pole<br>R. | 2.63[b]<br>2.58 | 2.65[bc]<br>2.56 | 2.71[c]<br>2.62 | 1.75<br>1.17 | .176<br>.310 | .241<br>.376 |
| L. Fusiform<br>R. | 2.65[a]<br>2.71[a] | 2.59[c]<br>2.64 | 2.66[ac]<br>2.72[a] | 7.98<br>10.04 | <.001<br><.001 | .003<br>.001 |
| L. Inferior parietal<br>R. | 2.43[a]<br>2.49[a] | 2.37<br>2.44 | 2.44[a]<br>2.51[a] | 14.09<br>7.11 | <.001<br>.001 | <.001<br>.006 |
| L. Inferior temporal<br>R. | 2.66<br>2.77[a] | 2.63[c]<br>2.70[c] | 2.70[c]<br>2.78[ac] | 4.59<br>7.33 | .011<br>.001 | .030<br>.005 |
| L. Insula<br>R. | 3.03<br>3.03[a] | 2.98<br>2.98[c] | 3.02<br>3.04[ac] | 3.35<br>2.87 | .036<br>.058 | .069<br>.096 |
| L. Isthmus cingulate<br>R. | 2.42<br>2.39[a] | 2.37<br>2.34[c] | 2.47<br>2.42[ac] | 7.10<br>5.34 | .001<br>.005 | .006<br>.019 |
| L. Lateral occipital<br>R. | 2.12[a]<br>2.20[a] | 2.07<br>2.14 | 2.13[a]<br>2.21[a] | 13.16<br>8.01 | <.001<br><.001 | <.001<br>.003 |
| L. Lateral orbitofrontal<br>R. | 2.62<br>2.53 | 2.61<br>2.49[c] | 2.64<br>2.55[c] | 0.05<br>3.97 | .954<br>.020 | .954<br>.046 |
| L. Lingual | 2.01[a] | 1.96 | 2.03[a] | 8.37 | <.001 | .003 |

| | | | | | | |
|---|---|---|---|---|---|---|
| R. | 2.02 | 1.99[c] | 2.05[c] | 5.37 | .005 | .019 |
| L. Medial orbitofrontal | 2.31[b] | 2.34[b] | 2.36 | 1.00 | .369 | .415 |
| R. | 2.22 | 2.22 | 2.24 | 0.21 | .813 | .821 |
| L. Middle temporal | 2.83[a] | 2.78 | 2.86[a] | 4.26 | .015 | .038 |
| R. | 2.86[a] | 2.79 | 2.87[a] | 8.66 | <.001 | .002 |
| L. Paracentral | 2.37 | 2.34[c] | 2.40[c] | 3.94 | .020 | .046 |
| R. | 2.39 | 2.36[c] | 2.41[c] | 4.68 | .010 | .029 |
| L. Parahippocampal | 2.70 | 2.64[c] | 2.73[c] | 1.41 | .246 | .313 |
| R. | 2.68 | 2.63[c] | 2.73[c] | 2.30 | .102 | .152 |
| L. Pars opercularis | 2.59 | 2.56 | 2.63 | 2.81 | .062 | .100 |
| R. | 2.56 | 2.52[c] | 2.58[c] | 3.19 | .042 | .078 |
| L. Pars orbitalis | 2.68 | 2.62[c] | 2.69[c] | 3.48 | .032 | .065 |
| R. | 2.57 | 2.54 | 2.57 | 1.13 | .324 | .380 |
| L. Pars triangularis | 2.46 | 2.42 | 2.50 | 4.72 | .010 | .028 |
| R. | 2.39 | 2.35[c] | 2.41[c] | 4.12 | .017 | .042 |
| L. Pericalcarine | 1.66 | 1.66 | 1.69 | 0.59 | .554 | .586 |
| R. | 1.68 | 1.66[c] | 1.70[c] | 1.72 | .180 | .243 |
| L. Postcentral | 2.13[a] | 2.07[c] | 2.13[ac] | 11.68 | <.001 | <.001 |
| R. | 2.09[a] | 2.04 | 2.11[a] | 10.48 | <.001 | .001 |
| L. Posterior cingulate | 2.47 | 2.43[c] | 2.48[c] | 2.13 | .121 | .173 |
| R. | 2.44[a] | 2.39[c] | 2.42[ac] | 4.82 | .009 | .027 |
| L. Precentral | 2.63[a] | 2.57[c] | 2.64[ac] | 5.80 | .003 | .016 |
| R. | 2.54 | 2.50[c] | 2.56[c] | 4.27 | .015 | .038 |
| L. Precuneus | 2.37 | 2.31 | 2.38 | 14.16 | <.001 | <.001 |
| R. | 2.38 | 2.35 | 2.40 | 4.32 | .014 | .037 |
| L. Rostral anterior cingulate | 2.79 | 2.76[c] | 2.83[c] | 1.72 | .181 | .243 |
| R. | 2.69 | 2.66 | 2.71 | 1.15 | .318 | .378 |
| L. Rostral middle frontal | 2.37[b] | 2.35[bc] | 2.41[c] | 2.27 | .105 | .154 |
| R. | 2.24[b] | 2.23[bc] | 2.28[c] | 2.06 | .130 | .183 |
| L. Superior frontal | 2.71 | 2.67 | 2.74 | 3.61 | .028 | .058 |
| R. | 2.65 | 2.61 | 2.68 | 5.50 | .004 | .018 |
| L. Superior parietal | 2.20[a] | 2.16 | 2.22[a] | 12.27 | <.001 | <.001 |
| R. | 2.20 | 2.17 | 2.23 | 6.12 | .002 | .013 |
| L. Superior temporal | 2.83[a] | 2.76[c] | 2.81[ac] | 6.38 | .002 | .010 |
| R. | 2.82[a] | 2.75[c] | 2.80[ac] | 7.84 | <.001 | .004 |
| L. Supramarginal | 2.55[a] | 2.50 | 2.57[a] | 8.78 | <.001 | .002 |
| R. | 2.57[a] | 2.53 | 2.59[a] | 6.03 | .003 | .013 |
| L. Temporal pole | 3.58 | 3.51[c] | 3.63[c] | 4.86 | .008 | .027 |

| | | | | | | |
|---|---|---|---|---|---|---|
| R. | 3.82[a] | 3.71[c] | 3.80[ac] | 3.09 | .047 | .084 |
| L. Transverse temporal<br>R. | 2.51[b]<br>2.52[a] | 2.39[b]<br>2.43[c] | 2.44<br>2.51[ac] | 10.75<br>7.01 | <.001<br>.001 | .001<br>.006 |
| ***Surface Area*** | | | | | | |
| L. Bankssts<br>R. | 1070<br>985 | 1051<br>963 | 1059<br>942 | 0.48<br>3.77 | .619<br>.024 | .651<br>.052 |
| L. Caudal anterior cingulate<br>R. | 660<br>817 | 647<br>797 | 645<br>815 | 0.36<br>0.17 | .695<br>.847 | .720<br>.851 |
| L. Caudal middle frontal<br>R. | 2425<br>2180 | 2356<br>2107 | 2321<br>2067 | 3.73<br>4.23 | .025<br>.015 | .054<br>.039 |
| L. Cuneus<br>R. | 1521<br>1567 | 1461<br>1519 | 1468<br>1529 | 3.89<br>3.02 | .021<br>.050 | .048<br>.087 |
| L. Entorhinal<br>R. | 422<br>359 | 418<br>355 | 413<br>346 | 0.34<br>1.28 | .712<br>.280 | .734<br>.348 |
| L. Frontal pole<br>R. | 206<br>290 | 200<br>285 | 202<br>286 | 0.64<br>0.78 | .528<br>.460 | .564<br>.504 |
| L. Fusiform<br>R. | 3282<br>3195 | 3209<br>3089 | 3240<br>3160 | 1.17<br>1.14 | .313<br>.321 | .376<br>.378 |
| L. Inferior parietal<br>R. | 4742[a]<br>5585 | 4554<br>5569 | 4599[a]<br>5555 | 2.92<br>1.27 | .055<br>.283 | .094<br>.350 |
| L. Inferior temporal<br>R. | 3311<br>3158 | 3187<br>3076 | 3204<br>3146 | 2.68<br>0.74 | .070<br>.477 | .112<br>.518 |
| L. Insula<br>R. | 2184<br>2253 | 2144<br>2210 | 2203<br>2273 | 1.53<br>0.92 | .217<br>.399 | .282<br>.445 |
| L. Isthmus cingulate<br>R. | 1049<br>942 | 1021<br>939 | 1013<br>936 | 2.9<br>0.82 | .057<br>.439 | .095<br>.485 |
| L. Lateral occipital<br>R. | 4897<br>4837[a] | 4805<br>4583 | 4821<br>4707[a] | 1.75<br>4.97 | .176<br>.007 | .241<br>.025 |
| L. Lateral orbitofrontal<br>R. | 2651[b]<br>2492 | 2502[b]<br>2416 | 2553<br>2417 | 10.74<br>5.55 | <.001<br>.004 | .001<br>.018 |
| L. Lingual<br>R. | 3157[a]<br>3209[a] | 3007<br>3044 | 3045[a]<br>3082[a] | 4.44<br>5.49 | .013<br>.005 | .034<br>.018 |
| L. Medial orbitofrontal<br>R. | 1821[b]<br>1744 | 1784[b]<br>1724 | 1739<br>1702 | 5.39<br>2.91 | .005<br>.056 | .019<br>.095 |
| L. Middle temporal<br>R. | 3182[ab]<br>3502[a] | 3017[b]<br>3350 | 3046[a]<br>3376[a] | 6.57<br>5.75 | .002<br>.004 | .009<br>.016 |
| L. Paracentral<br>R. | 1329<br>1500 | 1315<br>1466 | 1299<br>1468 | 2.07<br>1.03 | .128<br>.358 | .182<br>.406 |

| | | | | | | |
|---|---|---|---|---|---|---|
| L. Parahippocampal | 721 | 696 | 691 | 2.87 | .058 | .096 |
| R. | 700[a] | 667 | 672[a] | 3.37 | .036 | .068 |
| L. Pars opercularis | 1787[ab] | 1640[b] | 1694[a] | 8.60 | <.001 | .002 |
| R. | 1429 | 1358 | 1423 | 2.15 | .119 | .173 |
| L. Pars orbitalis | 648[a] | 612 | 629[a] | 5.51 | .004 | .018 |
| R. | 807[a] | 760[c] | 802[ac] | 6.96 | .001 | .006 |
| L. Pars triangularis | 1331 | 1278 | 1300 | 1.65 | .194 | .256 |
| R. | 1531 | 1502 | 1532 | 0.23 | .796 | .808 |
| L. Pericalcarine | 1444[a] | 1370 | 1383[a] | 4.02 | .019 | .045 |
| R. | 1611[ab] | 1527[b] | 1516[a] | 6.91 | .001 | .006 |
| L. Postcentral | 4244 | 4103 | 4125 | 2.88 | .058 | .096 |
| R. | 4001 | 3869 | 3895 | 3.44 | .033 | .065 |
| L. Posterior cingulate | 1190 | 1170 | 1178 | 0.70 | .498 | .534 |
| R. | 1240 | 1198 | 1225 | 1.29 | .277 | .347 |
| L. Precentral | 4835 | 4751 | 4765 | 2.02 | .134 | .187 |
| R. | 4920 | 4795 | 4869 | 1.68 | .188 | .250 |
| L. Precuneus | 3864 | 3745 | 3735 | 5.17 | .006 | .022 |
| R. | 3970 | 3864 | 3904 | 2.00 | .137 | .190 |
| L. Rostral anterior cingulate | 852 | 831 | 816 | 2.67 | .071 | .112 |
| R. | 719 | 686 | 682 | 2.49 | .085 | .13 |
| L. Rostral middle frontal | 5986 | 5839 | 5857 | 3.28 | .039 | .073 |
| R. | 6307 | 6150 | 6092 | 5.08 | .007 | .023 |
| L. Superior frontal | 7307 | 7136 | 7101 | 5.14 | .006 | .022 |
| R. | 7098 | 6937 | 6887 | 4.57 | .011 | .030 |
| L. Superior parietal | 5594[a] | 5395 | 5479[a] | 3.47 | .032 | .065 |
| R. | 5627 | 5487 | 5435 | 5.66 | .004 | .017 |
| L. Superior temporal | 3844[a] | 3718 | 3792[a] | 2.57 | .078 | .122 |
| R. | 3602 | 3575 | 3586 | 0.71 | .493 | .532 |
| L. Supramarginal | 3903 | 3820 | 3904 | 0.43 | .650 | .680 |
| R. | 3773 | 3636 | 3694 | 2.05 | .130 | .183 |
| L. Temporal pole | 491 | 479 | 483 | 1.16 | .314 | .376 |
| R. | 435 | 425 | 421 | 1.34 | .264 | .335 |
| L. Transverse temporal | 446 | 455 | 466 | 0.85 | .428 | .475 |
| R. | 334 | 330 | 336 | 0.24 | .783 | .799 |
| ***Subcortical Volume*** | | | | | | |
| L. Bankssts | 2627 | 2535 | 2627 | 0.76 | .470 | .513 |
| R. | 2501[a] | 2372 | 2391[a] | 3.67 | .027 | .057 |
| L. Caudal anterior cingulate | 1876 | 1796 | 1818 | 0.98 | .377 | .423 |
| R. | 2216 | 2123 | 2213 | 0.62 | .538 | .572 |

| | | | | | | |
|---|---|---|---|---|---|---|
| L. Caudal middle frontal | 6863 | 6567 | 6653 | 3.08 | .047 | .084 |
| R. | 6182[a] | 5802 | 5915[a] | 4.65 | .010 | .029 |
| L. Cuneus | 3159[a] | 2982 | 3087[a] | 3.45 | .033 | .065 |
| R. | 3273 | 3131 | 3258 | 2.13 | .120 | .173 |
| L. Entorhinal | 1928 | 1875 | 1930 | 1.03 | .358 | .406 |
| R. | 1845 | 1814 | 1793 | 1.30 | .275 | .347 |
| L. Frontal pole | 735 | 718 | 753 | 0.39 | .680 | .708 |
| R. | 1030 | 999 | 1041 | 1.08 | .339 | .389 |
| L. Fusiform | 10064[a] | 9610 | 9954[a] | 3.87 | .022 | .049 |
| R. | 9872[a] | 9256[c] | 9816[ac] | 5.55 | .004 | .018 |
| L. Inferior parietal | 12880[a] | 12048[c] | 12585[ac] | 7.66 | .001 | .004 |
| R. | 15339 | 14884 | 15402 | 1.12 | .329 | .381 |
| L. Inferior temporal | 10321[a] | 9845 | 10166[a] | 3.03 | .050 | .087 |
| R. | 10336[a] | 9800[c] | 10318[ac] | 3.77 | .024 | .052 |
| L. Insula | 6722[a] | 6496[c] | 6766[ac] | 3.51 | .031 | .064 |
| R. | 6962[a] | 6710[c] | 7034[ac] | 3.10 | .047 | .084 |
| L. Isthmus cingulate | 2735[a] | 2598[c] | 2718[ac] | 2.29 | .103 | .153 |
| R. | 2448 | 2366 | 2458 | 1.15 | .319 | .378 |
| L. Lateral occipital | 11536[a] | 10969[c] | 11371[ac] | 4.14 | .017 | .042 |
| R. | 11666 | 10736[c] | 11428[c] | 10.93 | <.001 | .001 |
| L. Lateral orbitofrontal | 7608 | 7163[c] | 7467[c] | 11.98 | <.001 | <.001 |
| R. | 7058[a] | 6745[c] | 6966[ac] | 7.97 | <.001 | .003 |
| L. Lingual | 6884[a] | 6449[c] | 6786[ac] | 5.73 | .004 | .016 |
| R. | 7014[a] | 6559[c] | 6895[ac] | 5.98 | .003 | .014 |
| L. Medial orbitofrontal | 4762 | 4740 | 4694 | 3.38 | .035 | .068 |
| R. | 4607 | 4580 | 4556 | 2.93 | .055 | .094 |
| L. Middle temporal | 10933 | 10146[c] | 10613[c] | 9.55 | <.001 | .001 |
| R. | 12132 | 11285[c] | 11752[c] | 11.74 | <.001 | <.001 |
| L. Paracentral | 3460 | 3379 | 3436 | 1.10 | .333 | .384 |
| R. | 3896 | 3744 | 3867 | 1.51 | .221 | .284 |
| L. Parahippocampal | 2281[a] | 2151 | 2237[a] | 2.79 | .063 | .101 |
| R. | 2152[a] | 2013[c] | 2104[ac] | 4.04 | .019 | .044 |
| L. Pars opercularis | 5240[a] | 4786[c] | 5094[ac] | 7.97 | <.001 | .003 |
| R. | 4123[a] | 3833[c] | 4133[ac] | 4.81 | .009 | .027 |
| L. Pars orbitalis | 2200 | 2036[c] | 2168[c] | 7.35 | .001 | .005 |
| R. | 2637 | 2448 | 2636 | 9.31 | <.001 | .001 |
| L. Pars triangularis | 3712[a] | 3497[c] | 3697[ac] | 2.30 | .101 | .152 |
| R. | 4194 | 4068[c] | 4288[c] | 0.32 | .728 | .746 |
| L. Pericalcarine | 2246 | 2139 | 2190 | 1.74 | .177 | .241 |

| | | | | | | |
|---|---|---|---|---|---|---|
| R. | 2570[a] | 2404 | 2455[a] | 3.96 | .020 | .046 |
| L. Postcentral<br>R. | 10184[a]<br>9383[a] | 9542[c]<br>8832[c] | 9911[ac]<br>9225[ac] | 6.94<br>6.96 | .001<br>.001 | .006<br>.006 |
| L. Posterior cingulate<br>R. | 3196<br>3305[a] | 3081<br>3085[c] | 3204<br>3234[ac] | 1.20<br>5.24 | .303<br>.006 | .371<br>.021 |
| L. Precentral<br>R. | 13889[a]<br>13636[a] | 13345[c]<br>13038[c] | 13780[ac]<br>13621[ac] | 3.10<br>4.09 | .046<br>.018 | .084<br>.043 |
| L. Precuneus<br>R. | 9950[a]<br>10119[a] | 9398[c]<br>9680[c] | 9717[ac]<br>10055[ac] | 8.24<br>4.57 | <.001<br>.011 | .003<br>.030 |
| L. Rostral anterior cingulate<br>R. | 2717<br>2241 | 2612<br>2119 | 2638<br>2148 | 2.51<br>2.84 | .083<br>.060 | .128<br>.097 |
| L. Rostral middle frontal<br>R. | 16104[a]<br>16371 | 15545[c]<br>15809 | 16128[ac]<br>16146 | 2.32<br>3.65 | .100<br>.027 | .150<br>.057 |
| L. Superior frontal<br>R. | 22570[a]<br>21701[a] | 21770<br>20893 | 22320[a]<br>21422[a] | 5.20<br>4.74 | .006<br>.009 | .021<br>.028 |
| L. Superior parietal<br>R. | 13684[a]<br>13701[a] | 12907[c]<br>13161 | 13554[ac]<br>13432[a] | 9.41<br>4.79 | <.001<br>.009 | .001<br>.027 |
| L. Superior temporal<br>R. | 12346[a]<br>11628 | 11721[c]<br>11242 | 12170[ac]<br>11532 | 5.94<br>3.43 | .003<br>.034 | .014<br>.065 |
| L. Supramarginal<br>R. | 11041[a]<br>10610[a] | 10551[c]<br>10027[c] | 11172[ac]<br>10466[ac] | 2.61<br>4.75 | .075<br>.009 | .118<br>.028 |
| L. Temporal pole<br>R. | 2517[a]<br>2420[a] | 2397[c]<br>2290 | 2521[ac]<br>2349[a] | 4.95<br>4.53 | .008<br>.012 | .025<br>.031 |
| L. Transverse temporal<br>R. | 1252<br>954 | 1203<br>909[c] | 1265<br>959[c] | 1.55<br>3.23 | .214<br>.041 | .280<br>.075 |

Note: L.=left; R.=right; bankssts = banks of the superior temporal sulcus; Groups that share a subscript reflect a significant ($p$<.05) pairwise comparison
